# Definitive crystal structure of 1,1′-bis­[1,2-dicarba-*closo*-dodeca­borane(11)]

**DOI:** 10.1107/S1600536814023897

**Published:** 2014-11-05

**Authors:** Wing Y. Man, Georgina M. Rosair, Alan J. Welch

**Affiliations:** a Institute of Chemical Sciences, Perkin Building, School of Engineering & Physical Sciences, Heriot Watt University, Edinburgh EH14 4AS, Scotland

**Keywords:** crystal structure, carboranes, 1,2-dicarba-*closo*-dodeca­borane(11), cage compounds

## Abstract

In the title compound, C_4_H_22_B_20_, the two {1,2-*closo*-C_2_B_10_H_11_} cages are linked across a centre of inversion with a C—C distance of 1.5339 (11) Å. By careful analysis of the structure, it is established that the non-linking cage C atom is equally disordered over cage vertices 2 and 3.

## Abstract 2

In the title compound, the two {1,2-*closo*-C_2_B_10_H_11_} cages are linked across a centre of inversion with a C—C distance of 1.5339 (11) Å. By careful analysis of the structure, it is established that the non-linking cage C atom is equally disordered over cage vertices 2 and 3.

## Chemical context   

The chemistry of single-cage carboranes is now regarded as a mature subject (Grimes, 2011 ▶) but that of bis­(carboranes), two discrete carborane units connected *via* a two-centre–two-electron bond, is far from fully developed. For bis­(carboranes) composed of two C_2_B_10_ icosa­hedra, there are several possible isomers of which 1,1′-bis­[1,2-dicarba-*closo*-dodeca­borane(11)] (Dupont & Hawthorne, 1964 ▶) is the best known. Aspects of the chemistry of this species have been partially explored (Hawthorne & Owen, 1971 ▶; Yanovsky *et al.*, 1979 ▶; Harwell *et al.*, 1996 ▶, 1997 ▶; Herzog *et al.*, 1999 ▶; Ellis *et al.*, 2010*a* ▶,*b* ▶) but there is still considerable scope for further development.
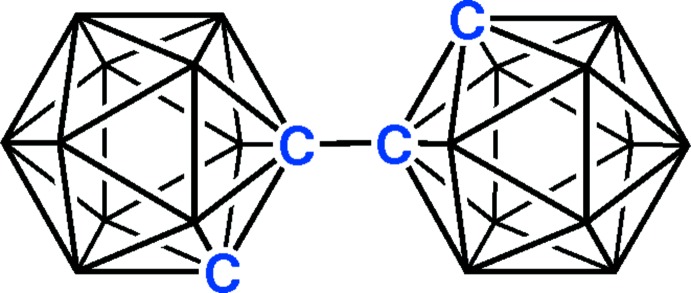


The two structural studies of 1,1′-bis­[1,2-dicarba-*closo*-dodeca­borane(11)] so far reported for which atomic coordin­ates are available (Hall *et al.*, 1965 ▶; Ren & Xie, 2008 ▶) agree that the overall mol­ecular structure is that of two 1,2-dicarba-*closo*-dodeca­borane(11) units linked *via* a C1—C1*A* bond across a centre of inversion. However they differ in their inter­pretation of the position of the non-linking carbon atom, C2 (and, by symmetry, C2*A*). In the earlier study, Hall *et al.* considered two models, one (Case I) in which C2 was disordered over two adjacent cage vertices and another (Case II) in which it was disordered over all five vertices to which C1 is connected, expressing a slight preference for the former model based on *R* factors, with supplementary evidence coming from inspection of temperature factors and the lengths of cage connectivities. In their later study, Ren & Xie considered only an ordered model, with C2 occupying one of the two C/B disordered sites in Case I of Hall *et al.*, but no justification for this model was given. The two crystals used by Hall *et al.* and by Ren & Xie are isomorphous, and both data sets were collected at room temperature.

We have recently described two new methods, which distinguish CH from BH vertices in carboranes and heterocarboranes, the *Vertex-to-Centroid Distance* (VCD) method (McAnaw *et al.*, 2013 ▶) and the *Boron–Hydrogen Distance* (BHD) method (McAnaw *et al.*, 2014 ▶). In the present communication, we apply these methods to a precise, low-temperature data set to unambiguously describe the crystal structure of the title compound, 1,1′-bis­[1,2-dicarba-*closo*-dodeca­borane(11)].

## Structural commentary   

**Figure 1:**
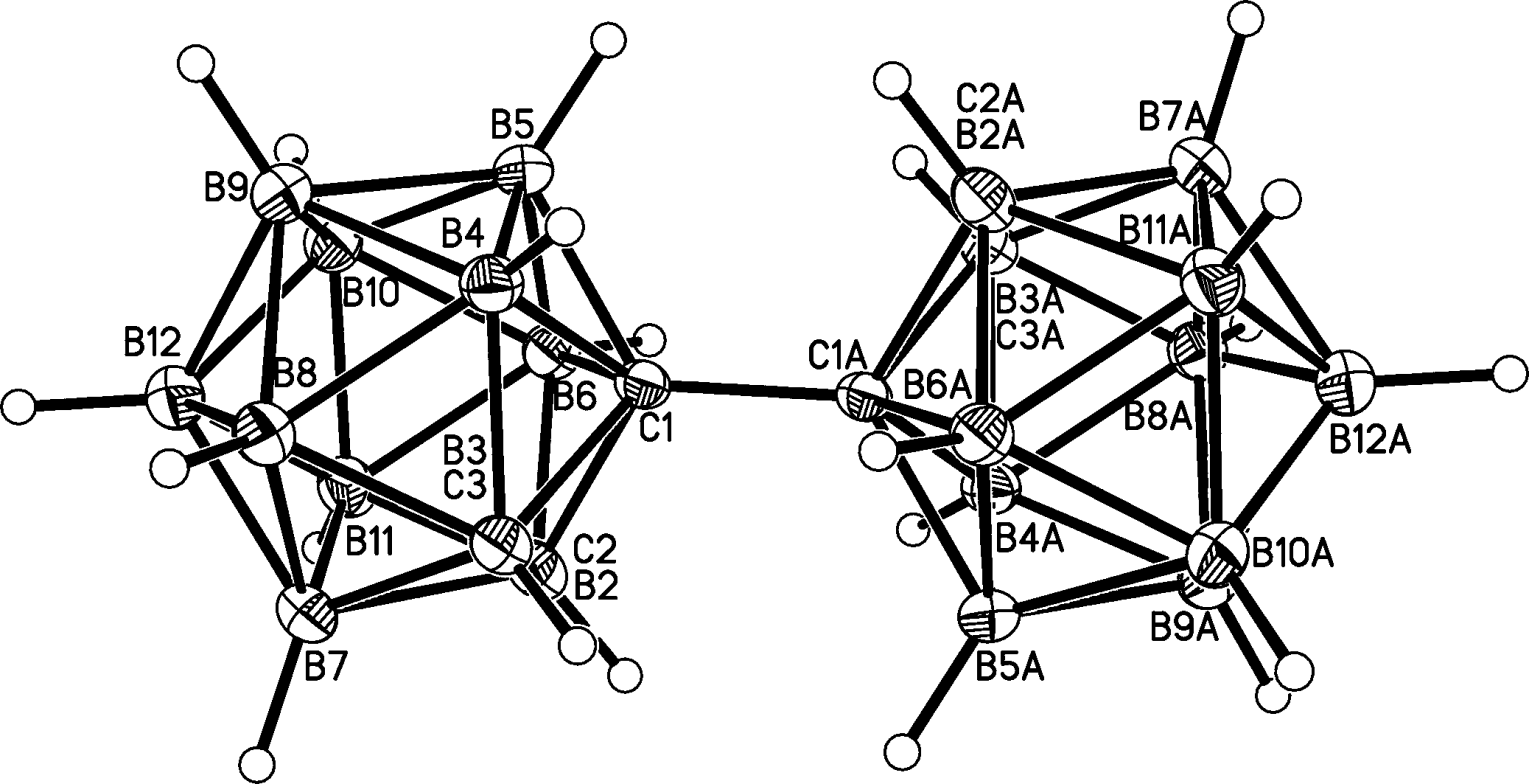
Perspective view of the title compound, with displacement ellipsoids drawn at the 50% probability level. The label suffix ‘A’ refers to the symmetry operation (−*x*, −*y* + 2, −*z* + 2).

Mol­ecules of 1,1′-bis­[1,2-dicarba-*closo*-dodeca­borane(11)] are composed of two {1,2-*closo*-C_2_B_10_H_11_} cages (the contents of one asymmetric fraction of the unit cell), linked across a crystallographic inversion centre by the C1–C1*A* bond [1.5339 (11) Å; symmetry code: (*A*) −*x*, −*y* + 2, −*z* + 2] (Fig. 1 ▶). The two cages are essentially co-linear, with B12⋯C1—C1*A* = 175.14 (5)°.

The crystals used in this determination are also isomorphous with those studied by Hall *et al.* (1965 ▶) and by Ren & Xie (2008 ▶), so comment on the positioning of the non-linking cage C atom in all three determinations is warranted. Using the *Vertex-to-Centroid Distance* (VCD) method (McAnaw *et al.*, 2013 ▶) to analyse our *Prostructure* (only the linking atom C1 identified as carbon with all other cage atoms described as boron and with H atoms allowed positional refinement), we conclude that the second cage C atom is statistically disordered over vertices 2 and 3 (Table 1 ▶). On assigning these positions as (essentially) 0.5C+0.5B and completing the refinement we note that all vertex–centroid distances barely change, confirming our contention (McAnaw *et al.*, 2013 ▶) that the conclusions from the VCD method are essentially independent of whether vertices have been refined as C or B and thus allowing the method to be applied to literature structures even if an incorrect C/B assignment has been made. Application of the VCD method to the structure of Hall *et al.* confirms that their partially disordered Case I model was correct, whilst application to the structure of Ren & Xie (which had the second C atom exclusively at vertex 3) shows that their model is incorrect. *Boron–Hydrogen Distance* (BHD) analysis (McAnaw *et al.*, 2014 ▶) of our structure (Table 2 ▶) also supports the conclusion that the non-linking C is disordered over vertices 2 and 3. The two shortest vertex–H distances in the *Prostructure* involve vertices 2 and 3, and when these vertices are assigned as (essentially) 0.5C+0.5B, the refined distances to H increase to values between those expected for 100% B and 100% C.

The final structure determined for 1,1′-bis­[1,2-dicarba-*closo*-dodeca­borane(11)] is the most precise to date. The e.s.d.’s on comparable mol­ecular parameters are *ca* half the magnitude of those of Hall *et al.* (which is nevertheless a remarkably good determination given the hardware used to collect data and the limited number of reflections measured) and *ca* a quarter of the magnitude of those of Ren & Xie. The present determination is the only one to have been carried out at low temperature (100 K).

The three C1—B distances span the range 1.7308 (9)–1.7427 (9) Å whilst the two C1—C/B connectivities are 1.6950 (8) and 1.6991 (8) Å. Of the remaining connectivities, C/B—C/B is shortest, 1.7215 (9) Å, C/B—B is inter­mediate, lying in the range 1.7353 (10)–1.7603 (9) Å, and B—B distances are the longest, spanning from 1.7775 (10) to 1.8015 (11) Å. The relative lengths of all of these connectivities are fully consistent with the fact that C has a smaller radius than B, which is the essential basis for the VCD method.

## Supra­molecular features   

The only H⋯H contact less than 2.40 Å is H3⋯H12*B* at 2.342 (13) Å [symmetry code: (*B*) −*x* + 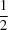, *y* + 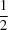, −*z* + 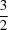]. Given that vertex B is 50% C and that CH units and BH units in carboranes are protonic and hydridic respectively, with the degree of hydridic character increasing with increasing distance from the C atoms, this might represent a weak di­hydrogen bond. The angles at H3 and H12*B* are 151.1 (7) and 123.2 (6)°, respectively.

## Database survey   

A search of the Cambridge Structural Database (Groom & Allen, 2014 ▶) for the 1,1′-bis­(1,2-dicarba-*closo*-dodeca­borane) unit using *Conquest* (Version 1.16) returns 13 hits. Of these, four are reported to be of the title compound (DOCBOR, DOCBOR01, DOCBOR02 and DOCBOR03). DOCBOR (Hall *et al.*, 1965 ▶) represents an early (room-temperature data, point detector, <2000 reflections collected) yet remarkably precise determination. DOCBOR01 (Swanson *et al.*, 1968 ▶) appears to be a powder diffraction study and certainly no resulting atomic coordinates are deposited. DOCBOR02 (Yang *et al.*, 1995 ▶) is ambiguously recorded in the Database; the actual mol­ecule which is the subject of the crystallographic study (compound **2** in the relevant paper) is [1-(3′-1′,2′-*closo*-C_2_B_10_H_11_)-2-*closo*-C_2_B_10_H_11_] with a C1—B3′ inter­cluster bond whereas 1,1′-bis­[1,2-dicarba-*closo*-dodeca­borane(11)] is [1-(1′-1′,2′-*closo*-C_2_B_10_H_11_)-2-*closo*-C_2_B_10_H_11_] with a C1—C1′ inter­cluster bond. Finally, the most recent published determination (DOCBOR03; Ren & Xie, 2008 ▶) involves data collected on a modern CCD-equipped diffractometer although also at room temperature.

Of the remaining nine hits revealed by *Conquest*, one (FASQAR; Herzog *et al.*, 1999 ▶) relates to an octa­methyl derivative of 1,1′-bis­[1,2-dicarba-*closo*-dodeca­borane(11)] and eight are concerned with species in which the mol­ecule has been deprotonated at the C2 and C2′ positions, with the resulting dianion complexing either a transition metal or a main-group element.

## Synthesis and crystallization   

The compound was prepared by the Cu^I^-mediated coupling of li­thia­ted *ortho*-carborane, a method first reported by Yang *et al.* (1995 ▶) for *para*-carborane and later used by Ren & Xie (2008 ▶) for the coupling of *ortho*-carborane. Purity was confirmed by elemental microanalysis, mass spectrometry and NMR spectroscopy, the last by comparison with the data of Yang *et al.* (1995 ▶). Single crystals for this study were afforded by cooling a solution of the compound in hexane to 243 K.

## Refinement   

Crystal data, data collection and structure refinement details are summarized in Table 3 ▶. The mol­ecule sits on a crystallographic centre of symmetry at the mid-point of the C1—C1*A* bond. Initially only the linking atom C1 was identified as carbon, with all other cage atoms described as boron and with H atoms allowed positional refinement. This model (the *Prostructure*) was refined and then analysed by both the VCD (McAnaw *et al.*, 2013 ▶) and the BHD (McAnaw *et al.*, 2014 ▶) methods. Both methods led to the same conclusion regarding the location of the second C atom, which was found to be disordered between positions 2 and 3. These vertices were assigned boron and carbon occupancies of 0.5, treated as tied variables. Refinement was completed with H atoms continuing to be refined positionally and with *U*_iso_(H) = 1.2*U*_eq_(C,B). At convergence, cage position 2 is [0.503 (9) C + 0.497 (9) B] and cage position 3 [0.497 (9) C + 0.503 (9) B]; effectively positions 2 and 3 are both 50% C + 50% B.

## Supplementary Material

Crystal structure: contains datablock(s) I. DOI: 10.1107/S1600536814023897/pk2536sup1.cif

Structure factors: contains datablock(s) I. DOI: 10.1107/S1600536814023897/pk2536Isup2.hkl

CCDC reference: 1031659

Additional supporting information:  crystallographic information; 3D view; checkCIF report

## Figures and Tables

**Table 1:** Vertex-to-centroid distances () in studies of 1,1-bis[1,2-dicarba-*closo*-dodecaborane(11)]

Vertex	Hall *et al.* (1965 ▶)	Ren Zie (2008 ▶)	This study (*Prostructure*)	This study (final structure)
1	1.5890(10)	1.590(2)	1.5969(8)	1.5975(6)
2	1.6274(13)	1.627(2)	1.6385(10)	1.6384(7)
3	1.6291(12)	1.632(2)	1.6420(9)	1.6418(7)
4	1.6893(13)	1.700(2)	1.7129(9)	1.7117(7)
5	1.6938(14)	1.692(2)	1.7069(9)	1.7054(7)
6	1.6817(13)	1.696(2)	1.7145(9)	1.7124(7)
7	1.6904(14)	1.694(3)	1.7085(9)	1.7086(7)
8	1.6839(15)	1.685(3)	1.7002(10)	1.7002(7)
9	1.6740(15)	1.681(3)	1.6920(10)	1.6920(7)
10	1.6717(15)	1.672(3)	1.6900(10)	1.6900(8)
11	1.6888(14)	1.685(3)	1.7020(10)	1.7019(8)
12	1.6657(16)	1.665(3)	1.6779(10)	1.6780(8)

**Table 2:** Vertex-to-H distances () in *Prostructure* and final structure of 1,1-bis[1,2-dicarba-*closo*-dodecaborane(11)]

Vertex	Distance (*Prostructure*)	Distance (final structure)
2	0.842(12)	1.030(9)
3	0.902(11)	1.006(9)
4	1.066(11)	1.083(9)
5	1.105(11)	1.094(8)
6	1.088(11)	1.096(8)
7	1.110(11)	1.089(9)
8	1.088(11)	1.069(9)
9	1.043(12)	1.080(9)
10	1.164(12)	1.108(9)
11	1.118(11)	1.096(9)
12	1.086(11)	1.108(9)

**Table 3:** Experimental details

Crystal data	
Chemical formula	C_4_H_22_B_20_
*M*_r_	286.41
Crystal system, space group	Monoclinic, *P*2_1_/*n*
Temperature (K)	100
*a*, *b*, *c* ()	7.0011(5), 9.7667(6), 12.4071(8)
()	90.375(3)
*V* (^3^)	848.35(10)
*Z*	2
Radiation type	Mo *K*
(mm^1^)	0.05
Crystal size (mm)	0.38 0.34 0.32

Data collection	
Diffractometer	Bruker APEXII CCD
Absorption correction	Multi-scan (*SADABS*; Bruker, 2008 ▶)
*T*_min_, *T*_max_	0.706, 0.747
No. of measured, independent and observed [*I* > 2(*I*)] reflections	22850, 3324, 2787
*R*_int_	0.029
(sin /)_max_ (^1^)	0.778

Refinement	
*R*[*F* ^2^ > 2(*F* ^2^)], *wR*(*F* ^2^), *S*	0.036, 0.106, 1.05
No. of reflections	3324
No. of parameters	143
H-atom treatment	Only H-atom coordinates refined
_max_, _min_ (e ^3^)	0.30, 0.22

Computer programs: *SAINT* and *APEX2* (Bruker, 2009 ▶), *SHELXS97* and *SHELXL2014* (Sheldrick, 2008 ▶) and *OLEX2* (Dolomanov *et al.*, 2009 ▶).

## supplementary crystallographic information

### Crystal data

**Table:**

C_4_H_22_B_20_	*F*(000) = 292
*M**_r_* = 286.41	*D*_x_ = 1.121 Mg m^−^^3^
Monoclinic, *P*2_1_/*n*	Mo *K*α radiation, λ = 0.71073 Å
*a* = 7.0011 (5) Å	Cell parameters from 7872 reflections
*b* = 9.7667 (6) Å	θ = 3.3–33.5°
*c* = 12.4071 (8) Å	µ = 0.05 mm^−^^1^
β = 90.375 (3)°	*T* = 100 K
*V* = 848.35 (10) Å^3^	BLOCK, colourless
*Z* = 2	0.38 × 0.34 × 0.32 mm

### Data collection

**Table:**

Bruker APEXII CCD diffractometer	3324 independent reflections
Radiation source: sealed tube	2787 reflections with *I* > 2σ(*I*)
Graphite monochromator	*R*_int_ = 0.029
φ and ω scans	θ_max_ = 33.6°, θ_min_ = 2.7°
Absorption correction: multi-scan (*SADABS*; Bruker, 2008)	*h* = −10→10
*T*_min_ = 0.706, *T*_max_ = 0.747	*k* = −14→15
22850 measured reflections	*l* = −19→19

### Refinement

**Table:**

Refinement on *F*^2^	0 restraints
Least-squares matrix: full	Hydrogen site location: difference Fourier map
*R*[*F*^2^ > 2σ(*F*^2^)] = 0.036	Only H-atom coordinates refined
*wR*(*F*^2^) = 0.106	*w* = 1/[σ^2^(*F*_o_^2^) + (0.0556*P*)^2^ + 0.1013*P*] where *P* = (*F*_o_^2^ + 2*F*_c_^2^)/3
*S* = 1.05	(Δ/σ)_max_ < 0.001
3324 reflections	Δρ_max_ = 0.30 e Å^−^^3^
143 parameters	Δρ_min_ = −0.22 e Å^−^^3^

### Special details

**Table:**

Experimental. Absorption correction: SADABS-2008/1 (Bruker, 2008) was used for absorption correction. wR2(int) was 0.0508 before and 0.0428 after correction. The Ratio of minimum to maximum transmission is 0.9456. The λ/2 correction factor is 0.0015.
Geometry. All e.s.d.'s (except the e.s.d. in the dihedral angle between two l.s. planes) are estimated using the full covariance matrix. The cell e.s.d.'s are taken into account individually in the estimation of e.s.d.'s in distances, angles and torsion angles; correlations between e.s.d.'s in cell parameters are only used when they are defined by crystal symmetry. An approximate (isotropic) treatment of cell e.s.d.'s is used for estimating e.s.d.'s involving l.s. planes.

### Fractional atomic coordinates and isotropic or equivalent isotropic displacement parameters (Å^2^)

**Table:**

	*x*	*y*	*z*	*U*_iso_*/*U*_eq_	Occ. (<1)
C1	−0.00588 (8)	0.94081 (6)	0.95949 (4)	0.01437 (11)	
B2	−0.07903 (9)	0.98070 (7)	0.83283 (5)	0.01927 (14)	0.497 (9)
H2	−0.1071 (12)	1.0828 (10)	0.8194 (7)	0.023*	
B3	0.15654 (9)	0.94099 (7)	0.85833 (5)	0.01846 (13)	0.503 (9)
H3	0.2474 (12)	1.0207 (9)	0.8580 (7)	0.022*	
B4	0.17020 (10)	0.81469 (7)	0.95931 (5)	0.01763 (13)	
H4	0.2854 (12)	0.8236 (9)	1.0176 (7)	0.021*	
B5	−0.07164 (10)	0.77626 (7)	0.99536 (5)	0.01763 (13)	
H5	−0.1033 (12)	0.7563 (9)	1.0801 (7)	0.021*	
B6	−0.22796 (9)	0.88235 (7)	0.91629 (5)	0.01762 (13)	
H6	−0.3531 (12)	0.9333 (9)	0.9506 (7)	0.021*	
B7	0.04606 (10)	0.87930 (7)	0.74140 (5)	0.01979 (14)	
H7	0.0862 (12)	0.9264 (9)	0.6652 (7)	0.024*	
B8	0.20135 (10)	0.77266 (7)	0.82037 (6)	0.01995 (14)	
H8	0.3405 (12)	0.7451 (10)	0.7928 (7)	0.024*	
B9	0.05645 (11)	0.66684 (7)	0.90524 (6)	0.02070 (14)	
H9	0.1038 (12)	0.5664 (9)	0.9302 (7)	0.025*	
B10	−0.19002 (11)	0.70885 (7)	0.87886 (6)	0.02095 (14)	
H10	−0.3091 (13)	0.6353 (10)	0.8892 (7)	0.025*	
B11	−0.19577 (10)	0.84046 (8)	0.77730 (6)	0.02052 (14)	
H11	−0.3151 (12)	0.8578 (10)	0.7213 (7)	0.025*	
B12	−0.02108 (11)	0.70670 (7)	0.77061 (6)	0.02187 (14)	
H12	−0.0230 (12)	0.6296 (10)	0.7051 (7)	0.026*	
C2	−0.07903 (9)	0.98070 (7)	0.83283 (5)	0.01927 (14)	0.503 (9)
C3	0.15654 (9)	0.94099 (7)	0.85833 (5)	0.01846 (13)	0.497 (9)

### Atomic displacement parameters (Å^2^)

**Table:**

	*U*^11^	*U*^22^	*U*^33^	*U*^12^	*U*^13^	*U*^23^
C1	0.0157 (2)	0.0151 (2)	0.0123 (2)	−0.00095 (18)	0.00111 (17)	0.00166 (16)
B2	0.0196 (3)	0.0240 (3)	0.0142 (3)	−0.0027 (2)	0.0005 (2)	−0.0006 (2)
B3	0.0220 (3)	0.0187 (3)	0.0147 (3)	0.0011 (2)	0.0012 (2)	0.0010 (2)
B4	0.0191 (3)	0.0170 (3)	0.0169 (3)	0.0020 (2)	0.0016 (2)	0.0020 (2)
B5	0.0208 (3)	0.0155 (3)	0.0166 (3)	−0.0018 (2)	0.0022 (2)	0.0021 (2)
B6	0.0171 (3)	0.0188 (3)	0.0169 (3)	−0.0023 (2)	0.0000 (2)	0.0001 (2)
B7	0.0240 (3)	0.0212 (3)	0.0141 (3)	−0.0009 (2)	0.0019 (2)	0.0001 (2)
B8	0.0222 (3)	0.0195 (3)	0.0181 (3)	0.0015 (2)	0.0040 (2)	−0.0002 (2)
B9	0.0262 (3)	0.0162 (3)	0.0198 (3)	0.0004 (2)	0.0036 (2)	0.0007 (2)
B10	0.0244 (3)	0.0183 (3)	0.0202 (3)	−0.0044 (2)	0.0017 (2)	−0.0017 (2)
B11	0.0225 (3)	0.0222 (3)	0.0168 (3)	−0.0026 (2)	−0.0017 (2)	−0.0013 (2)
B12	0.0273 (3)	0.0198 (3)	0.0185 (3)	−0.0025 (3)	0.0023 (2)	−0.0022 (2)
C2	0.0196 (3)	0.0240 (3)	0.0142 (3)	−0.0027 (2)	0.0005 (2)	−0.0006 (2)
C3	0.0220 (3)	0.0187 (3)	0.0147 (3)	0.0011 (2)	0.0012 (2)	0.0010 (2)

### Geometric parameters (Å, º)

**Table:**

C1—C1^i^	1.5339 (11)	B6—H6	1.096 (8)
C1—B2	1.6950 (8)	B6—B10	1.7775 (10)
C1—B3	1.6991 (8)	B6—B11	1.7881 (10)
C1—B4	1.7427 (9)	B6—C2	1.7596 (9)
C1—B5	1.7308 (9)	B7—H7	1.089 (9)
C1—B6	1.7378 (9)	B7—B8	1.7924 (10)
C1—C2	1.6950 (8)	B7—B11	1.7941 (10)
C1—C3	1.6991 (8)	B7—B12	1.7877 (10)
B2—H2	1.030 (9)	B7—C2	1.7457 (9)
B2—B6	1.7596 (9)	B7—C3	1.7468 (9)
B2—B7	1.7457 (9)	B8—H8	1.069 (9)
B2—B11	1.7353 (10)	B8—B9	1.7944 (10)
B2—C3	1.7215 (9)	B8—B12	1.7912 (11)
B3—H3	1.006 (9)	B8—C3	1.7392 (10)
B3—B4	1.7603 (9)	B9—H9	1.080 (9)
B3—B7	1.7468 (9)	B9—B10	1.8015 (11)
B3—B8	1.7392 (10)	B9—B12	1.7956 (10)
B3—C2	1.7215 (9)	B10—H10	1.108 (9)
B4—H4	1.083 (9)	B10—B11	1.8003 (10)
B4—B5	1.7937 (10)	B10—B12	1.7956 (10)
B4—B8	1.7869 (10)	B11—H11	1.096 (9)
B4—B9	1.7784 (10)	B11—B12	1.7918 (11)
B4—C3	1.7603 (9)	B11—C2	1.7353 (10)
B5—H5	1.094 (8)	B12—H12	1.108 (9)
B5—B6	1.7944 (10)	C2—H2	1.030 (9)
B5—B9	1.7915 (10)	C3—H3	1.006 (9)
B5—B10	1.7871 (10)		
			
C1^i^—C1—B2	116.67 (6)	C2—B7—B3	59.07 (4)
C1^i^—C1—B3	116.75 (6)	C2—B7—H7	117.2 (5)
C1^i^—C1—B4	119.91 (6)	C2—B7—B8	106.23 (5)
C1^i^—C1—B5	123.00 (5)	C2—B7—B11	58.69 (4)
C1^i^—C1—B6	119.59 (6)	C2—B7—B12	105.64 (5)
C1^i^—C1—C2	116.67 (6)	C3—B7—H7	117.4 (5)
C1^i^—C1—C3	116.75 (6)	C3—B7—B8	58.85 (4)
B2—C1—B4	111.76 (4)	C3—B7—B11	106.28 (5)
B2—C1—B5	111.87 (4)	C3—B7—B12	105.81 (5)
B2—C1—B6	61.66 (4)	B3—B8—B4	59.88 (4)
B2—C1—C3	60.96 (4)	B3—B8—B7	59.27 (4)
B3—C1—B4	61.51 (4)	B3—B8—H8	119.4 (5)
B3—C1—B5	111.80 (4)	B3—B8—B9	106.37 (5)
B3—C1—B6	111.98 (4)	B3—B8—B12	105.98 (5)
B5—C1—B4	62.18 (4)	B4—B8—B7	108.45 (5)
B5—C1—B6	62.31 (4)	B4—B8—H8	118.9 (5)
B6—C1—B4	113.51 (5)	B4—B8—B9	59.55 (4)
C2—C1—B3	60.96 (4)	B4—B8—B12	107.69 (5)
C2—C1—B4	111.76 (4)	B7—B8—H8	121.5 (5)
C2—C1—B5	111.87 (4)	B7—B8—B9	108.19 (5)
C2—C1—B6	61.66 (4)	B9—B8—H8	124.3 (5)
C3—C1—B4	61.51 (4)	B12—B8—B7	59.85 (4)
C3—C1—B5	111.80 (4)	B12—B8—H8	126.2 (5)
C3—C1—B6	111.98 (4)	B12—B8—B9	60.10 (4)
C1—B2—H2	115.4 (5)	C3—B8—B4	59.88 (4)
C1—B2—B6	60.37 (4)	C3—B8—B7	59.27 (4)
C1—B2—B7	108.80 (5)	C3—B8—H8	119.4 (5)
C1—B2—B11	108.99 (5)	C3—B8—B9	106.37 (5)
C1—B2—C3	59.64 (4)	C3—B8—B12	105.98 (5)
B6—B2—H2	120.7 (5)	B4—B9—B5	60.32 (4)
B7—B2—H2	122.7 (5)	B4—B9—B8	60.02 (4)
B7—B2—B6	111.99 (5)	B4—B9—H9	119.6 (5)
B11—B2—H2	127.7 (5)	B4—B9—B10	108.04 (5)
B11—B2—B6	61.54 (4)	B4—B9—B12	107.87 (5)
B11—B2—B7	62.04 (4)	B5—B9—B8	108.05 (5)
C3—B2—H2	115.4 (5)	B5—B9—H9	121.1 (5)
C3—B2—B6	109.85 (5)	B5—B9—B10	59.65 (4)
C3—B2—B7	60.50 (4)	B5—B9—B12	107.56 (5)
C3—B2—B11	110.08 (5)	B8—B9—H9	121.2 (5)
C1—B3—H3	115.5 (5)	B8—B9—B10	107.86 (5)
C1—B3—B4	60.47 (4)	B8—B9—B12	59.86 (4)
C1—B3—B7	108.56 (5)	B10—B9—H9	123.4 (5)
C1—B3—B8	108.78 (5)	B12—B9—H9	123.6 (5)
C1—B3—C2	59.40 (3)	B12—B9—B10	59.89 (4)
B4—B3—H3	120.9 (5)	B5—B10—B9	59.89 (4)
B7—B3—H3	122.7 (5)	B5—B10—H10	119.4 (5)
B7—B3—B4	111.79 (5)	B5—B10—B11	108.13 (5)
B8—B3—H3	128.0 (5)	B5—B10—B12	107.76 (5)
B8—B3—B4	61.40 (4)	B6—B10—B5	60.45 (4)
B8—B3—B7	61.88 (4)	B6—B10—B9	108.32 (5)
C2—B3—H3	115.5 (5)	B6—B10—H10	118.3 (5)
C2—B3—B4	109.64 (5)	B6—B10—B11	59.97 (4)
C2—B3—B7	60.44 (4)	B6—B10—B12	107.87 (5)
C2—B3—B8	109.72 (5)	B9—B10—H10	123.5 (5)
C1—B4—B3	58.03 (3)	B11—B10—B9	107.86 (5)
C1—B4—H4	117.8 (5)	B11—B10—H10	122.0 (5)
C1—B4—B5	58.58 (4)	B12—B10—B9	59.89 (4)
C1—B4—B8	104.74 (4)	B12—B10—H10	125.3 (5)
C1—B4—B9	105.03 (5)	B12—B10—B11	59.77 (4)
C1—B4—C3	58.03 (3)	B2—B11—B6	59.90 (4)
B3—B4—H4	117.1 (5)	B2—B11—B7	59.26 (4)
B3—B4—B5	106.09 (5)	B2—B11—B10	106.10 (5)
B3—B4—B8	58.72 (4)	B2—B11—H11	119.0 (5)
B3—B4—B9	106.16 (5)	B2—B11—B12	105.90 (5)
B5—B4—H4	123.4 (5)	B6—B11—B7	108.43 (5)
B8—B4—H4	124.6 (5)	B6—B11—B10	59.38 (4)
B8—B4—B5	108.29 (5)	B6—B11—H11	118.4 (5)
B9—B4—H4	130.3 (5)	B6—B11—B12	107.57 (5)
B9—B4—B5	60.20 (4)	B7—B11—B10	107.93 (5)
B9—B4—B8	60.44 (4)	B7—B11—H11	121.8 (5)
C3—B4—H4	117.1 (5)	B10—B11—H11	124.6 (5)
C3—B4—B5	106.09 (5)	B12—B11—B7	59.81 (4)
C3—B4—B8	58.72 (4)	B12—B11—B10	59.98 (4)
C3—B4—B9	106.16 (5)	B12—B11—H11	126.9 (5)
C1—B5—B4	59.23 (3)	C2—B11—B6	59.90 (4)
C1—B5—H5	117.9 (5)	C2—B11—B7	59.26 (4)
C1—B5—B6	59.04 (4)	C2—B11—B10	106.10 (5)
C1—B5—B9	104.97 (4)	C2—B11—H11	119.0 (5)
C1—B5—B10	104.88 (4)	C2—B11—B12	105.90 (5)
B4—B5—H5	118.3 (4)	B7—B12—B8	60.11 (4)
B4—B5—B6	108.43 (4)	B7—B12—B9	108.34 (5)
B6—B5—H5	120.1 (5)	B7—B12—B10	108.42 (5)
B9—B5—B4	59.47 (4)	B7—B12—B11	60.16 (4)
B9—B5—H5	126.8 (5)	B7—B12—H12	119.6 (5)
B9—B5—B6	108.01 (5)	B8—B12—B9	60.04 (4)
B10—B5—B4	108.00 (5)	B8—B12—B10	108.27 (5)
B10—B5—H5	128.1 (5)	B8—B12—B11	108.30 (5)
B10—B5—B6	59.51 (4)	B8—B12—H12	120.2 (5)
B10—B5—B9	60.45 (4)	B9—B12—H12	122.5 (5)
C1—B6—B2	57.98 (3)	B10—B12—B9	60.22 (4)
C1—B6—B5	58.66 (3)	B10—B12—H12	123.5 (5)
C1—B6—H6	116.6 (5)	B11—B12—B9	108.50 (5)
C1—B6—B10	105.00 (5)	B11—B12—B10	60.24 (4)
C1—B6—B11	104.74 (4)	B11—B12—H12	121.6 (5)
C1—B6—C2	57.98 (3)	C1—C2—H2	115.4 (5)
B2—B6—B5	105.98 (4)	C1—C2—B3	59.64 (4)
B2—B6—H6	117.3 (5)	C1—C2—B6	60.37 (4)
B2—B6—B10	106.05 (5)	C1—C2—B7	108.80 (5)
B2—B6—B11	58.56 (4)	C1—C2—B11	108.99 (5)
B5—B6—H6	122.4 (4)	B3—C2—H2	115.4 (5)
B10—B6—B5	60.04 (4)	B3—C2—B6	109.85 (5)
B10—B6—H6	131.0 (5)	B3—C2—B7	60.50 (4)
B10—B6—B11	60.65 (4)	B3—C2—B11	110.08 (5)
B11—B6—B5	108.35 (5)	B6—C2—H2	120.7 (5)
B11—B6—H6	125.8 (4)	B7—C2—H2	122.7 (5)
C2—B6—B5	105.98 (4)	B7—C2—B6	111.99 (5)
C2—B6—H6	117.3 (5)	B11—C2—H2	127.7 (5)
C2—B6—B10	106.05 (5)	B11—C2—B6	61.54 (4)
C2—B6—B11	58.56 (4)	B11—C2—B7	62.04 (4)
B2—B7—H7	117.2 (5)	C1—C3—B2	59.40 (3)
B2—B7—B8	106.23 (5)	C1—C3—H3	115.5 (5)
B2—B7—B11	58.69 (4)	C1—C3—B4	60.47 (4)
B2—B7—B12	105.64 (5)	C1—C3—B7	108.56 (5)
B2—B7—C3	59.07 (4)	C1—C3—B8	108.78 (5)
B3—B7—H7	117.4 (5)	B2—C3—H3	115.5 (5)
B3—B7—B8	58.85 (4)	B2—C3—B4	109.64 (5)
B3—B7—B11	106.28 (5)	B2—C3—B7	60.44 (4)
B3—B7—B12	105.81 (5)	B2—C3—B8	109.72 (5)
B8—B7—H7	124.1 (5)	B4—C3—H3	120.9 (5)
B8—B7—B11	108.15 (5)	B7—C3—H3	122.7 (5)
B11—B7—H7	123.6 (5)	B7—C3—B4	111.79 (5)
B12—B7—H7	130.0 (5)	B8—C3—H3	128.0 (5)
B12—B7—B8	60.04 (4)	B8—C3—B4	61.40 (4)
B12—B7—B11	60.03 (4)	B8—C3—B7	61.88 (4)
			
C1^i^—C1—B2—B6	−110.91 (7)	B6—B2—B11—B7	−140.34 (5)
C1^i^—C1—B2—B7	143.93 (6)	B6—B2—B11—B10	−38.74 (5)
C1^i^—C1—B2—B11	−150.06 (6)	B6—B2—B11—B12	−101.40 (5)
C1^i^—C1—B2—C3	107.28 (6)	B6—B2—C3—C1	−34.85 (4)
C1^i^—C1—B3—B4	111.21 (7)	B6—B2—C3—B4	0.16 (6)
C1^i^—C1—B3—B7	−143.73 (6)	B6—B2—C3—B7	104.67 (5)
C1^i^—C1—B3—B8	150.53 (6)	B6—B2—C3—B8	65.83 (6)
C1^i^—C1—B3—C2	−107.17 (7)	B6—B5—B9—B4	101.20 (5)
C1^i^—C1—B4—B3	−106.17 (7)	B6—B5—B9—B8	63.45 (6)
C1^i^—C1—B4—B5	114.01 (7)	B6—B5—B9—B10	−37.09 (4)
C1^i^—C1—B4—B8	−143.31 (6)	B6—B5—B9—B12	0.25 (6)
C1^i^—C1—B4—B9	153.92 (6)	B6—B5—B10—B9	138.27 (5)
C1^i^—C1—B4—C3	−106.17 (7)	B6—B5—B10—B11	37.71 (4)
C1^i^—C1—B5—B4	−109.25 (7)	B6—B5—B10—B12	100.89 (5)
C1^i^—C1—B5—B6	108.91 (7)	B6—B10—B11—B2	38.98 (5)
C1^i^—C1—B5—B9	−148.80 (6)	B6—B10—B11—B7	101.22 (5)
C1^i^—C1—B5—B10	148.40 (6)	B6—B10—B11—B12	138.33 (5)
C1^i^—C1—B6—B2	106.28 (7)	B6—B10—B11—C2	38.98 (5)
C1^i^—C1—B6—B5	−114.16 (7)	B6—B10—B12—B7	0.18 (7)
C1^i^—C1—B6—B10	−153.94 (6)	B6—B10—B12—B8	63.87 (6)
C1^i^—C1—B6—B11	143.08 (6)	B6—B10—B12—B9	101.23 (5)
C1^i^—C1—B6—C2	106.28 (7)	B6—B10—B12—B11	−37.21 (5)
C1^i^—C1—C2—B3	107.28 (6)	B6—B11—B12—B7	−101.50 (5)
C1^i^—C1—C2—B6	−110.91 (7)	B6—B11—B12—B8	−64.15 (6)
C1^i^—C1—C2—B7	143.93 (6)	B6—B11—B12—B9	−0.51 (7)
C1^i^—C1—C2—B11	−150.06 (6)	B6—B11—B12—B10	36.88 (5)
C1^i^—C1—C3—B2	−107.17 (7)	B6—B11—C2—C1	38.62 (4)
C1^i^—C1—C3—B4	111.21 (7)	B6—B11—C2—B3	102.30 (5)
C1^i^—C1—C3—B7	−143.73 (6)	B6—B11—C2—B7	140.34 (5)
C1^i^—C1—C3—B8	150.53 (6)	B7—B2—B6—C1	99.79 (5)
C1—B2—B6—B5	−35.18 (4)	B7—B2—B6—B5	64.61 (6)
C1—B2—B6—B10	−97.92 (5)	B7—B2—B6—B10	1.87 (6)
C1—B2—B6—B11	−137.24 (5)	B7—B2—B6—B11	−37.44 (5)
C1—B2—B7—B8	0.34 (6)	B7—B2—B11—B6	140.34 (5)
C1—B2—B7—B11	102.03 (5)	B7—B2—B11—B10	101.61 (5)
C1—B2—B7—B12	63.05 (6)	B7—B2—B11—B12	38.94 (5)
C1—B2—B7—C3	−36.28 (4)	B7—B2—C3—C1	−139.51 (5)
C1—B2—B11—B6	38.62 (4)	B7—B2—C3—B4	−104.51 (5)
C1—B2—B11—B7	−101.72 (5)	B7—B2—C3—B8	−38.84 (4)
C1—B2—B11—B10	−0.12 (6)	B7—B3—B4—C1	−99.64 (5)
C1—B2—B11—B12	−62.78 (6)	B7—B3—B4—B5	−64.68 (6)
C1—B2—C3—B4	35.00 (4)	B7—B3—B4—B8	37.26 (5)
C1—B2—C3—B7	139.51 (5)	B7—B3—B4—B9	−1.74 (6)
C1—B2—C3—B8	100.68 (5)	B7—B3—B8—B4	−140.40 (5)
C1—B3—B4—B5	34.96 (4)	B7—B3—B8—B9	−101.77 (5)
C1—B3—B4—B8	136.90 (5)	B7—B3—B8—B12	−38.91 (4)
C1—B3—B4—B9	97.90 (5)	B7—B3—C2—C1	139.51 (5)
C1—B3—B7—B8	−101.86 (5)	B7—B3—C2—B6	104.67 (5)
C1—B3—B7—B11	−0.17 (6)	B7—B3—C2—B11	38.71 (5)
C1—B3—B7—B12	−62.90 (6)	B7—B8—B9—B4	−101.14 (5)
C1—B3—B7—C2	36.13 (4)	B7—B8—B9—B5	−63.25 (6)
C1—B3—B8—B4	−38.89 (4)	B7—B8—B9—B10	−0.21 (6)
C1—B3—B8—B7	101.51 (5)	B7—B8—B9—B12	36.99 (5)
C1—B3—B8—B9	−0.26 (6)	B7—B8—B12—B9	−138.63 (5)
C1—B3—B8—B12	62.60 (6)	B7—B8—B12—B10	−101.19 (5)
C1—B3—C2—B6	−34.85 (4)	B7—B8—B12—B11	−37.38 (5)
C1—B3—C2—B7	−139.51 (5)	B7—B8—C3—C1	101.51 (5)
C1—B3—C2—B11	−100.80 (5)	B7—B8—C3—B2	38.20 (4)
C1—B4—B5—B6	33.95 (4)	B7—B8—C3—B4	140.40 (5)
C1—B4—B5—B9	134.43 (5)	B7—B11—B12—B8	37.35 (5)
C1—B4—B5—B10	96.94 (5)	B7—B11—B12—B9	100.99 (5)
C1—B4—B8—B3	36.82 (4)	B7—B11—B12—B10	138.38 (5)
C1—B4—B8—B7	1.54 (6)	B7—B11—C2—C1	−101.72 (5)
C1—B4—B8—B9	−99.16 (5)	B7—B11—C2—B3	−38.04 (5)
C1—B4—B8—B12	−61.75 (6)	B7—B11—C2—B6	−140.34 (5)
C1—B4—B8—C3	36.82 (4)	B8—B3—B4—C1	−136.90 (5)
C1—B4—B9—B5	−39.12 (4)	B8—B3—B4—B5	−101.94 (5)
C1—B4—B9—B8	98.66 (5)	B8—B3—B4—B9	−39.00 (5)
C1—B4—B9—B10	−1.98 (6)	B8—B3—B7—B11	101.70 (5)
C1—B4—B9—B12	61.32 (6)	B8—B3—B7—B12	38.96 (5)
C1—B4—C3—B2	−34.58 (4)	B8—B3—B7—C2	137.99 (5)
C1—B4—C3—B7	−99.64 (5)	B8—B3—C2—C1	100.68 (5)
C1—B4—C3—B8	−136.90 (5)	B8—B3—C2—B6	65.83 (6)
C1—B5—B6—B2	34.89 (4)	B8—B3—C2—B7	−38.84 (4)
C1—B5—B6—B10	134.50 (5)	B8—B3—C2—B11	−0.12 (6)
C1—B5—B6—B11	96.43 (5)	B8—B4—B5—C1	−96.44 (5)
C1—B5—B6—C2	34.89 (4)	B8—B4—B5—B6	−62.49 (6)
C1—B5—B9—B4	39.43 (4)	B8—B4—B5—B9	37.99 (5)
C1—B5—B9—B8	1.68 (6)	B8—B4—B5—B10	0.51 (6)
C1—B5—B9—B10	−98.86 (5)	B8—B4—B9—B5	−137.78 (5)
C1—B5—B9—B12	−61.52 (6)	B8—B4—B9—B10	−100.64 (5)
C1—B5—B10—B6	−39.26 (4)	B8—B4—B9—B12	−37.34 (5)
C1—B5—B10—B9	99.01 (5)	B8—B4—C3—C1	136.90 (5)
C1—B5—B10—B11	−1.55 (6)	B8—B4—C3—B2	102.33 (5)
C1—B5—B10—B12	61.62 (6)	B8—B4—C3—B7	37.26 (5)
C1—B6—B10—B5	39.10 (4)	B8—B7—B11—B2	98.33 (5)
C1—B6—B10—B9	1.76 (6)	B8—B7—B11—B6	62.75 (6)
C1—B6—B10—B11	−98.72 (5)	B8—B7—B11—B10	−0.10 (6)
C1—B6—B10—B12	−61.59 (6)	B8—B7—B11—B12	−37.29 (5)
C1—B6—B11—B2	−36.53 (4)	B8—B7—B11—C2	98.33 (5)
C1—B6—B11—B7	−1.21 (6)	B8—B7—B12—B9	37.11 (5)
C1—B6—B11—B10	99.16 (5)	B8—B7—B12—B10	100.93 (5)
C1—B6—B11—B12	62.01 (6)	B8—B7—B12—B11	138.36 (5)
C1—B6—B11—C2	−36.53 (4)	B8—B7—C2—C1	0.34 (6)
C1—B6—C2—B3	34.55 (4)	B8—B7—C2—B3	36.63 (4)
C1—B6—C2—B7	99.79 (5)	B8—B7—C2—B6	−64.46 (6)
C1—B6—C2—B11	137.24 (5)	B8—B7—C2—B11	−101.69 (5)
B2—C1—B4—B5	−104.06 (5)	B8—B7—C3—C1	−101.86 (5)
B2—C1—B4—B8	−1.38 (6)	B8—B7—C3—B2	−137.99 (5)
B2—C1—B4—B9	−64.14 (5)	B8—B7—C3—B4	−37.06 (5)
B2—C1—B4—C3	35.77 (4)	B8—B9—B10—B5	−100.87 (5)
B2—C1—B5—B4	103.88 (5)	B8—B9—B10—B6	−63.29 (6)
B2—C1—B5—B6	−37.96 (4)	B8—B9—B10—B11	0.15 (6)
B2—C1—B5—B9	64.33 (6)	B8—B9—B10—B12	37.18 (5)
B2—C1—B5—B10	1.53 (6)	B8—B9—B12—B7	−37.14 (5)
B2—C1—B6—B5	139.56 (5)	B8—B9—B12—B10	−138.31 (5)
B2—C1—B6—B10	99.79 (5)	B8—B9—B12—B11	−100.91 (5)
B2—C1—B6—B11	36.80 (4)	B9—B4—B5—C1	−134.43 (5)
B2—C1—C3—B4	−141.62 (5)	B9—B4—B5—B6	−100.48 (5)
B2—C1—C3—B7	−36.56 (4)	B9—B4—B5—B10	−37.49 (5)
B2—C1—C3—B8	−102.30 (5)	B9—B4—B8—B3	135.98 (5)
B2—B6—B10—B5	99.49 (5)	B9—B4—B8—B7	100.70 (5)
B2—B6—B10—B9	62.15 (6)	B9—B4—B8—B12	37.40 (5)
B2—B6—B10—B11	−38.33 (4)	B9—B4—B8—C3	135.98 (5)
B2—B6—B10—B12	−1.21 (6)	B9—B4—C3—C1	97.90 (5)
B2—B6—B11—B7	35.32 (5)	B9—B4—C3—B2	63.32 (6)
B2—B6—B11—B10	135.69 (5)	B9—B4—C3—B7	−1.74 (6)
B2—B6—B11—B12	98.55 (5)	B9—B4—C3—B8	−39.00 (5)
B2—B7—B8—B4	−1.19 (6)	B9—B5—B6—C1	−96.99 (5)
B2—B7—B8—B9	61.89 (6)	B9—B5—B6—B2	−62.10 (6)
B2—B7—B8—B12	98.99 (5)	B9—B5—B6—B10	37.51 (5)
B2—B7—B8—C3	−36.72 (4)	B9—B5—B6—B11	−0.56 (6)
B2—B7—B11—B6	−35.59 (5)	B9—B5—B6—C2	−62.10 (6)
B2—B7—B11—B10	−98.43 (5)	B9—B5—B10—B6	−138.27 (5)
B2—B7—B11—B12	−135.62 (5)	B9—B5—B10—B11	−100.56 (5)
B2—B7—B12—B8	−100.01 (5)	B9—B5—B10—B12	−37.39 (5)
B2—B7—B12—B9	−62.90 (6)	B9—B8—B12—B7	138.63 (5)
B2—B7—B12—B10	0.92 (6)	B9—B8—B12—B10	37.44 (5)
B2—B7—B12—B11	38.35 (4)	B9—B8—B12—B11	101.25 (5)
B2—B7—C3—C1	36.13 (4)	B9—B8—C3—C1	−0.26 (6)
B2—B7—C3—B4	100.92 (5)	B9—B8—C3—B2	−63.56 (6)
B2—B7—C3—B8	137.99 (5)	B9—B8—C3—B4	38.64 (5)
B2—B11—B12—B7	−38.68 (4)	B9—B8—C3—B7	−101.77 (5)
B2—B11—B12—B8	−1.33 (6)	B9—B10—B11—B2	−62.27 (6)
B2—B11—B12—B9	62.31 (6)	B9—B10—B11—B6	−101.25 (5)
B2—B11—B12—B10	99.69 (5)	B9—B10—B11—B7	−0.03 (6)
B3—C1—B4—B5	−139.82 (5)	B9—B10—B11—B12	37.09 (5)
B3—C1—B4—B8	−37.14 (4)	B9—B10—B11—C2	−62.27 (6)
B3—C1—B4—B9	−99.91 (5)	B9—B10—B12—B7	−101.04 (5)
B3—C1—B5—B4	37.64 (4)	B9—B10—B12—B8	−37.36 (5)
B3—C1—B5—B6	−104.20 (5)	B9—B10—B12—B11	−138.44 (5)
B3—C1—B5—B9	−1.91 (6)	B10—B5—B6—C1	−134.50 (5)
B3—C1—B5—B10	−64.71 (6)	B10—B5—B6—B2	−99.61 (5)
B3—C1—B6—B5	103.91 (5)	B10—B5—B6—B11	−38.07 (5)
B3—C1—B6—B10	64.13 (5)	B10—B5—B6—C2	−99.61 (5)
B3—C1—B6—B11	1.15 (6)	B10—B5—B9—B4	138.29 (5)
B3—C1—B6—C2	−35.66 (5)	B10—B5—B9—B8	100.55 (5)
B3—C1—C2—B6	141.81 (5)	B10—B5—B9—B12	37.34 (5)
B3—C1—C2—B7	36.65 (4)	B10—B6—B11—B2	−135.69 (5)
B3—C1—C2—B11	102.66 (5)	B10—B6—B11—B7	−100.36 (5)
B3—B4—B5—C1	−34.72 (4)	B10—B6—B11—B12	−37.14 (5)
B3—B4—B5—B6	−0.77 (6)	B10—B6—B11—C2	−135.69 (5)
B3—B4—B5—B9	99.71 (5)	B10—B6—C2—C1	−97.92 (5)
B3—B4—B5—B10	62.22 (6)	B10—B6—C2—B3	−63.36 (6)
B3—B4—B8—B7	−35.28 (5)	B10—B6—C2—B7	1.87 (6)
B3—B4—B8—B9	−135.98 (5)	B10—B6—C2—B11	39.32 (5)
B3—B4—B8—B12	−98.57 (5)	B10—B9—B12—B7	101.17 (5)
B3—B4—B9—B5	−99.59 (5)	B10—B9—B12—B8	138.31 (5)
B3—B4—B9—B8	38.20 (4)	B10—B9—B12—B11	37.40 (5)
B3—B4—B9—B10	−62.44 (6)	B10—B11—B12—B7	−138.38 (5)
B3—B4—B9—B12	0.86 (6)	B10—B11—B12—B8	−101.02 (5)
B3—B7—B8—B4	35.53 (5)	B10—B11—B12—B9	−37.39 (5)
B3—B7—B8—B9	98.61 (5)	B10—B11—C2—C1	−0.12 (6)
B3—B7—B8—B12	135.71 (5)	B10—B11—C2—B3	63.57 (6)
B3—B7—B11—B6	0.87 (6)	B10—B11—C2—B6	−38.74 (5)
B3—B7—B11—B10	−61.97 (6)	B10—B11—C2—B7	101.61 (5)
B3—B7—B11—B12	−99.17 (5)	B11—B2—B6—C1	137.24 (5)
B3—B7—B11—C2	36.46 (4)	B11—B2—B6—B5	102.05 (5)
B3—B7—B12—B8	−38.40 (4)	B11—B2—B6—B10	39.32 (5)
B3—B7—B12—B9	−1.30 (6)	B11—B2—B7—B8	−101.69 (5)
B3—B7—B12—B10	62.53 (6)	B11—B2—B7—B12	−38.99 (5)
B3—B7—B12—B11	99.96 (5)	B11—B2—B7—C3	−138.32 (5)
B3—B7—C2—C1	−36.28 (4)	B11—B2—C3—C1	−100.80 (5)
B3—B7—C2—B6	−101.08 (5)	B11—B2—C3—B4	−65.80 (6)
B3—B7—C2—B11	−138.32 (5)	B11—B2—C3—B7	38.71 (5)
B3—B8—B9—B4	−38.79 (4)	B11—B2—C3—B8	−0.12 (6)
B3—B8—B9—B5	−0.91 (6)	B11—B6—B10—B5	137.82 (5)
B3—B8—B9—B10	62.14 (6)	B11—B6—B10—B9	100.48 (5)
B3—B8—B9—B12	99.33 (5)	B11—B6—B10—B12	37.12 (5)
B3—B8—B12—B7	38.64 (4)	B11—B6—C2—C1	−137.24 (5)
B3—B8—B12—B9	−99.99 (5)	B11—B6—C2—B3	−102.68 (5)
B3—B8—B12—B10	−62.55 (6)	B11—B6—C2—B7	−37.44 (5)
B3—B8—B12—B11	1.26 (6)	B11—B7—B8—B3	−98.42 (5)
B4—C1—B2—B6	105.82 (5)	B11—B7—B8—B4	−62.89 (6)
B4—C1—B2—B7	0.67 (6)	B11—B7—B8—B9	0.19 (6)
B4—C1—B2—B11	66.68 (6)	B11—B7—B8—B12	37.28 (5)
B4—C1—B2—C3	−35.98 (5)	B11—B7—B8—C3	−98.42 (5)
B4—C1—B3—B7	105.05 (5)	B11—B7—B12—B8	−138.36 (5)
B4—C1—B3—B8	39.32 (5)	B11—B7—B12—B9	−101.25 (5)
B4—C1—B3—C2	141.62 (5)	B11—B7—B12—B10	−37.43 (5)
B4—C1—B5—B6	−141.84 (5)	B11—B7—C2—C1	102.03 (5)
B4—C1—B5—B9	−39.55 (4)	B11—B7—C2—B3	138.32 (5)
B4—C1—B5—B10	−102.35 (5)	B11—B7—C2—B6	37.24 (5)
B4—C1—B6—B2	−102.98 (5)	B11—B7—C3—C1	−0.17 (6)
B4—C1—B6—B5	36.58 (4)	B11—B7—C3—B2	−36.29 (5)
B4—C1—B6—B10	−3.20 (6)	B11—B7—C3—B4	64.63 (6)
B4—C1—B6—B11	−66.18 (6)	B11—B7—C3—B8	101.70 (5)
B4—C1—B6—C2	−102.98 (5)	B11—B10—B12—B7	37.39 (5)
B4—C1—C2—B3	−35.98 (5)	B11—B10—B12—B8	101.08 (5)
B4—C1—C2—B6	105.82 (5)	B11—B10—B12—B9	138.44 (5)
B4—C1—C2—B7	0.67 (6)	B12—B7—B8—B3	−135.71 (5)
B4—C1—C2—B11	66.68 (6)	B12—B7—B8—B4	−100.17 (5)
B4—C1—C3—B2	141.62 (5)	B12—B7—B8—B9	−37.10 (5)
B4—C1—C3—B7	105.05 (5)	B12—B7—B8—C3	−135.71 (5)
B4—C1—C3—B8	39.32 (5)	B12—B7—B11—B2	135.62 (5)
B4—B3—B7—B8	−37.06 (5)	B12—B7—B11—B6	100.04 (5)
B4—B3—B7—B11	64.63 (6)	B12—B7—B11—B10	37.19 (5)
B4—B3—B7—B12	1.90 (6)	B12—B7—B11—C2	135.62 (5)
B4—B3—B7—C2	100.92 (5)	B12—B7—C2—C1	63.05 (6)
B4—B3—B8—B7	140.40 (5)	B12—B7—C2—B3	99.33 (5)
B4—B3—B8—B9	38.64 (5)	B12—B7—C2—B6	−1.75 (6)
B4—B3—B8—B12	101.49 (5)	B12—B7—C2—B11	−38.99 (5)
B4—B3—C2—C1	35.00 (4)	B12—B7—C3—C1	−62.90 (6)
B4—B3—C2—B6	0.16 (6)	B12—B7—C3—B2	−99.02 (5)
B4—B3—C2—B7	−104.51 (5)	B12—B7—C3—B4	1.90 (6)
B4—B3—C2—B11	−65.80 (6)	B12—B7—C3—B8	38.96 (5)
B4—B5—B6—C1	−34.03 (4)	B12—B8—B9—B4	−138.12 (5)
B4—B5—B6—B2	0.86 (6)	B12—B8—B9—B5	−100.24 (5)
B4—B5—B6—B10	100.47 (5)	B12—B8—B9—B10	−37.19 (5)
B4—B5—B6—B11	62.40 (6)	B12—B8—C3—C1	62.60 (6)
B4—B5—B6—C2	0.86 (6)	B12—B8—C3—B2	−0.71 (6)
B4—B5—B9—B8	−37.75 (5)	B12—B8—C3—B4	101.49 (5)
B4—B5—B9—B10	−138.29 (5)	B12—B8—C3—B7	−38.91 (4)
B4—B5—B9—B12	−100.95 (5)	B12—B9—B10—B5	−138.05 (5)
B4—B5—B10—B6	−101.22 (5)	B12—B9—B10—B6	−100.47 (5)
B4—B5—B10—B9	37.06 (4)	B12—B9—B10—B11	−37.03 (5)
B4—B5—B10—B11	−63.50 (6)	B12—B10—B11—B2	−99.36 (5)
B4—B5—B10—B12	−0.33 (6)	B12—B10—B11—B6	−138.33 (5)
B4—B8—B9—B5	37.88 (5)	B12—B10—B11—B7	−37.12 (5)
B4—B8—B9—B10	100.93 (5)	B12—B10—B11—C2	−99.36 (5)
B4—B8—B9—B12	138.12 (5)	B12—B11—C2—C1	−62.78 (6)
B4—B8—B12—B7	101.47 (5)	B12—B11—C2—B3	0.90 (6)
B4—B8—B12—B9	−37.16 (5)	B12—B11—C2—B6	−101.40 (5)
B4—B8—B12—B10	0.28 (7)	B12—B11—C2—B7	38.94 (5)
B4—B8—B12—B11	64.09 (6)	C2—C1—B3—B4	−141.62 (5)
B4—B8—C3—C1	−38.89 (4)	C2—C1—B3—B7	−36.56 (4)
B4—B8—C3—B2	−102.20 (5)	C2—C1—B3—B8	−102.30 (5)
B4—B8—C3—B7	−140.40 (5)	C2—C1—B4—B3	35.77 (4)
B4—B9—B10—B5	−37.44 (4)	C2—C1—B4—B5	−104.06 (5)
B4—B9—B10—B6	0.14 (6)	C2—C1—B4—B8	−1.38 (6)
B4—B9—B10—B11	63.58 (6)	C2—C1—B4—B9	−64.14 (5)
B4—B9—B10—B12	100.61 (5)	C2—C1—B5—B4	103.88 (5)
B4—B9—B12—B7	0.27 (7)	C2—C1—B5—B6	−37.96 (4)
B4—B9—B12—B8	37.41 (5)	C2—C1—B5—B9	64.33 (6)
B4—B9—B12—B10	−100.90 (5)	C2—C1—B5—B10	1.53 (6)
B4—B9—B12—B11	−63.50 (6)	C2—C1—B6—B5	139.56 (5)
B5—C1—B2—B6	38.23 (4)	C2—C1—B6—B10	99.79 (5)
B5—C1—B2—B7	−66.93 (6)	C2—C1—B6—B11	36.80 (4)
B5—C1—B2—B11	−0.91 (6)	C2—B3—B4—C1	−34.58 (4)
B5—C1—B2—C3	−103.58 (5)	C2—B3—B4—B5	0.39 (6)
B5—C1—B3—B4	−37.92 (5)	C2—B3—B4—B8	102.33 (5)
B5—C1—B3—B7	67.14 (6)	C2—B3—B4—B9	63.32 (6)
B5—C1—B3—B8	1.40 (6)	C2—B3—B7—B8	−137.99 (5)
B5—C1—B3—C2	103.70 (5)	C2—B3—B7—B11	−36.29 (5)
B5—C1—B4—B3	139.82 (5)	C2—B3—B7—B12	−99.02 (5)
B5—C1—B4—B8	102.68 (5)	C2—B3—B8—B4	−102.20 (5)
B5—C1—B4—B9	39.91 (4)	C2—B3—B8—B7	38.20 (4)
B5—C1—B4—C3	139.82 (5)	C2—B3—B8—B9	−63.56 (6)
B5—C1—B6—B2	−139.56 (5)	C2—B3—B8—B12	−0.71 (6)
B5—C1—B6—B10	−39.78 (4)	C2—B6—B10—B5	99.49 (5)
B5—C1—B6—B11	−102.76 (5)	C2—B6—B10—B9	62.15 (6)
B5—C1—B6—C2	−139.56 (5)	C2—B6—B10—B11	−38.33 (4)
B5—C1—C2—B3	−103.58 (5)	C2—B6—B10—B12	−1.21 (6)
B5—C1—C2—B6	38.23 (4)	C2—B6—B11—B7	35.32 (5)
B5—C1—C2—B7	−66.93 (6)	C2—B6—B11—B10	135.69 (5)
B5—C1—C2—B11	−0.91 (6)	C2—B6—B11—B12	98.55 (5)
B5—C1—C3—B2	103.70 (5)	C2—B7—B8—B3	−36.72 (4)
B5—C1—C3—B4	−37.92 (5)	C2—B7—B8—B4	−1.19 (6)
B5—C1—C3—B7	67.14 (6)	C2—B7—B8—B9	61.89 (6)
B5—C1—C3—B8	1.40 (6)	C2—B7—B8—B12	98.99 (5)
B5—B4—B8—B3	98.09 (5)	C2—B7—B11—B6	−35.59 (5)
B5—B4—B8—B7	62.81 (6)	C2—B7—B11—B10	−98.43 (5)
B5—B4—B8—B9	−37.89 (5)	C2—B7—B11—B12	−135.62 (5)
B5—B4—B8—B12	−0.49 (6)	C2—B7—B12—B8	−100.01 (5)
B5—B4—B8—C3	98.09 (5)	C2—B7—B12—B9	−62.90 (6)
B5—B4—B9—B8	137.78 (5)	C2—B7—B12—B10	0.92 (6)
B5—B4—B9—B10	37.15 (4)	C2—B7—B12—B11	38.35 (4)
B5—B4—B9—B12	100.44 (5)	C2—B11—B12—B7	−38.68 (4)
B5—B4—C3—C1	34.96 (4)	C2—B11—B12—B8	−1.33 (6)
B5—B4—C3—B2	0.39 (6)	C2—B11—B12—B9	62.31 (6)
B5—B4—C3—B7	−64.68 (6)	C2—B11—B12—B10	99.69 (5)
B5—B4—C3—B8	−101.94 (5)	C3—C1—B2—B6	141.81 (5)
B5—B6—B10—B9	−37.34 (4)	C3—C1—B2—B7	36.65 (4)
B5—B6—B10—B11	−137.82 (5)	C3—C1—B2—B11	102.66 (5)
B5—B6—B10—B12	−100.70 (5)	C3—C1—B4—B5	−139.82 (5)
B5—B6—B11—B2	−97.88 (5)	C3—C1—B4—B8	−37.14 (4)
B5—B6—B11—B7	−62.56 (6)	C3—C1—B4—B9	−99.91 (5)
B5—B6—B11—B10	37.80 (5)	C3—C1—B5—B4	37.64 (4)
B5—B6—B11—B12	0.66 (6)	C3—C1—B5—B6	−104.20 (5)
B5—B6—B11—C2	−97.88 (5)	C3—C1—B5—B9	−1.91 (6)
B5—B6—C2—C1	−35.18 (4)	C3—C1—B5—B10	−64.71 (6)
B5—B6—C2—B3	−0.63 (6)	C3—C1—B6—B2	−35.66 (5)
B5—B6—C2—B7	64.61 (6)	C3—C1—B6—B5	103.91 (5)
B5—B6—C2—B11	102.05 (5)	C3—C1—B6—B10	64.13 (5)
B5—B9—B10—B6	37.58 (4)	C3—C1—B6—B11	1.15 (6)
B5—B9—B10—B11	101.02 (5)	C3—B2—B6—C1	34.55 (4)
B5—B9—B10—B12	138.05 (5)	C3—B2—B6—B5	−0.63 (6)
B5—B9—B12—B7	63.94 (6)	C3—B2—B6—B10	−63.36 (6)
B5—B9—B12—B8	101.08 (5)	C3—B2—B6—B11	−102.68 (5)
B5—B9—B12—B10	−37.23 (5)	C3—B2—B7—B8	36.63 (4)
B5—B9—B12—B11	0.16 (7)	C3—B2—B7—B11	138.32 (5)
B5—B10—B11—B2	1.05 (6)	C3—B2—B7—B12	99.33 (5)
B5—B10—B11—B6	−37.93 (4)	C3—B2—B11—B6	102.30 (5)
B5—B10—B11—B7	63.29 (6)	C3—B2—B11—B7	−38.04 (5)
B5—B10—B11—B12	100.41 (5)	C3—B2—B11—B10	63.57 (6)
B5—B10—B11—C2	1.05 (6)	C3—B2—B11—B12	0.90 (6)
B5—B10—B12—B7	−63.66 (6)	C3—B4—B5—C1	−34.72 (4)
B5—B10—B12—B8	0.03 (7)	C3—B4—B5—B6	−0.77 (6)
B5—B10—B12—B9	37.39 (5)	C3—B4—B5—B9	99.71 (5)
B5—B10—B12—B11	−101.05 (5)	C3—B4—B5—B10	62.22 (6)
B6—C1—B2—B7	−105.16 (5)	C3—B4—B8—B7	−35.28 (5)
B6—C1—B2—B11	−39.15 (5)	C3—B4—B8—B9	−135.98 (5)
B6—C1—B2—C3	−141.81 (5)	C3—B4—B8—B12	−98.57 (5)
B6—C1—B3—B4	−105.69 (5)	C3—B4—B9—B5	−99.59 (5)
B6—C1—B3—B7	−0.63 (6)	C3—B4—B9—B8	38.20 (4)
B6—C1—B3—B8	−66.37 (6)	C3—B4—B9—B10	−62.44 (6)
B6—C1—B3—C2	35.93 (5)	C3—B4—B9—B12	0.86 (6)
B6—C1—B4—B3	103.20 (5)	C3—B7—B8—B4	35.53 (5)
B6—C1—B4—B5	−36.63 (4)	C3—B7—B8—B9	98.61 (5)
B6—C1—B4—B8	66.05 (6)	C3—B7—B8—B12	135.71 (5)
B6—C1—B4—B9	3.29 (6)	C3—B7—B11—B2	36.46 (4)
B6—C1—B4—C3	103.20 (5)	C3—B7—B11—B6	0.87 (6)
B6—C1—B5—B4	141.84 (5)	C3—B7—B11—B10	−61.97 (6)
B6—C1—B5—B9	102.29 (5)	C3—B7—B11—B12	−99.17 (5)
B6—C1—B5—B10	39.49 (4)	C3—B7—B12—B8	−38.40 (4)
B6—C1—C2—B3	−141.81 (5)	C3—B7—B12—B9	−1.30 (6)
B6—C1—C2—B7	−105.16 (5)	C3—B7—B12—B10	62.53 (6)
B6—C1—C2—B11	−39.15 (5)	C3—B7—B12—B11	99.96 (5)
B6—C1—C3—B2	35.93 (5)	C3—B8—B9—B4	−38.79 (4)
B6—C1—C3—B4	−105.69 (5)	C3—B8—B9—B5	−0.91 (6)
B6—C1—C3—B7	−0.63 (6)	C3—B8—B9—B10	62.14 (6)
B6—C1—C3—B8	−66.37 (6)	C3—B8—B9—B12	99.33 (5)
B6—B2—B7—B8	−64.46 (6)	C3—B8—B12—B7	38.64 (4)
B6—B2—B7—B11	37.24 (5)	C3—B8—B12—B9	−99.99 (5)
B6—B2—B7—B12	−1.75 (6)	C3—B8—B12—B10	−62.55 (6)
B6—B2—B7—C3	−101.08 (5)	C3—B8—B12—B11	1.26 (6)

Symmetry code: (i) −*x*, −*y*+2, −*z*+2.
